# Ultrasound Real-Time Tissue Elastography Improves the Diagnostic Performance of the ACR Thyroid Imaging Reporting and Data System in Differentiating Malignant from Benign Thyroid Nodules: A Summary of 1525 Thyroid Nodules

**DOI:** 10.1155/2020/1749351

**Published:** 2020-04-14

**Authors:** Shufang Pei, Bin Zhang, Shuzhen Cong, Juanjuan Liu, Suqing Wu, Yuhao Dong, Lu Zhang, Shuixing Zhang

**Affiliations:** ^1^Department of Ultrasound, Guangdong Provincial People's Hospital, Guangdong Academy of Medical Sciences, Guangzhou, Guangdong, China; ^2^Department of Radiology, The First Affiliated Hospital, Jinan University, Guangzhou, Guangdong, China; ^3^The Second School of Clinical Medicine, Southern Medical University, Guangzhou, Guangdong, China; ^4^Department of Radiology, Guangdong Provincial People's Hospital, Guangdong Academy of Medical Sciences, Guangzhou, Guangdong, China

## Abstract

**Background:**

To explore the correlation between the ultrasound elasticity score (ES) of real-time tissue elastography (RTE) and the malignant risk stratification of the Thyroid Imaging Reporting and Data System (TI-RADS) and to evaluate the added value of RTE to TI-RADS in differentiating malignant nodules from benign ones.

**Methods:**

A total of 1,498 patients (885 women and 613 men; mean age of 43.5 ± 12.4 years) with 1,525 confirmed thyroid nodules (*D* = maximum diameter, *D* ≤ 2.5 cm) confirmed by fine-needle aspiration (FNA) and/or surgery were included. The nodules were divided into four groups based on their sizes (*D* ≤ 0.5 cm, 0.5 < *D* ≤ 1.0 cm, 1.0 < *D* ≤ 2.0 cm, and 2.0 < *D* ≤ 2.5 cm). We assigned an ES of RTE and malignant risk stratification of the TI-RADS category to each nodule. The correlation between the ES of RTE and the malignant risk stratification of TI-RADS category was analyzed by the Spearman's rank correlation. The diagnostic performances of RTE, TI-RADS, and their combination were compared by the receiver operator characteristic (ROC) analysis.

**Results:**

The ES of RTE and the malignant risk stratification of TI-RADS showed a strong correlation in the size intervals of 0.5 < *D* ≤ 1.0 cm, 1.0 < *D* ≤ 2.0 cm, and 2.0 < *D* ≤ 2.5 cm (*r* = 0.768, 0.711, and 0.743, respectively). The diagnostic performance of their combination for each size interval was always better than RTE or TI-RADS alone (for all, *P* < 0.001).

**Conclusions:**

Overall, The ES of RTE was strongly correlated with the malignant risk stratification of TI-RADS. The diagnostic performance of the combination of RTE and TI-RADS outperformed RTE or TI-RADS alone. Therefore, RTE may be an adjunctive tool to the current TI-RADS system for differentiating malignant from benign thyroid nodules.

## 1. Introduction

Thyroid nodule disease is common worldwide, and its incidence has grown rapidly over the past decade [1]. With the application of ultrasound (US) technology in thyroid scans, the number of thyroid nodules detected through nonpalpation has increased, reaching 20–67% of the general population [2]. Ahn et al. [3, 4] reported that the application of US technology in thyroid examination has resulted in a dramatic increase in the detection rate of thyroid cancer in South Korea, causing great concern among the population. However, the proportion of malignant nodules is 5–10% among all thyroid nodules [5], which means that only a small portion of nodules require surgery, and most nodules only require regular follow-up. Therefore, the major clinical challenge is to reliably differentiate malignant nodules that need to be treated surgically from the majority benign nodules that do not require surgery, which emphasizes the importance of reliable and highly accurate diagnostic methods that can differentiate malignant from benign nodules on reducing overdiagnosis and overtreatment.

At present, various technical approaches, such as US, computed tomography (CT), magnetic resonance imaging (MRI), positron emission tomography (PET), fine-needle aspiration (FNA), and genetic testing, are available for differentiating malignant from benign thyroid nodules. Among these, the conventional US is the most widely used and preferred adjunctive diagnostic tool because of the noninvasive, convenient, affordable, and nonradioactive characteristics. However, US also has several limitations in differentiating malignant from benign thyroid nodules. The multiple structures of nodules could lead to complex images and overlapping features of benign and malignant nodules, which would reduce the accuracy and specificity of US. To overcome these difficulties and find more effective diagnostic methods is particularly important.

In May 2017, the American College of Radiology (ACR) launched a classification system-Thyroid Imaging Reporting and Data System (TI-RADS) [6], in which all thyroid nodules are divided into five categories: 1, 2, 3, 4, and 5. Each category was given a different malignant risk, the malignant risk of TI-RADS 1, TI-RADS 2, TI-RADS 3, TI-RADS 4, and TI-RADS 5 was 2%, 2%, 5%, 5–20%, and more than 20%, respectively. Doctors treat the nodules differently based on this classification. In the process of evaluating thyroid nodules, we found that the application value of ACR TI-RADS was considerable, which helped to reduce the biopsy rate of thyroid nodules. However, it is possible for some thyroid nodules, especially small nodules, may have a broader management boundary and miss the suspicious nodules that should be paid attention to. For example, according to the existing evaluation indicators, a large part of thyroid nodules was classified as TI-RADS 4. Such nodules often only show solid component, hypoechoic, and without other common malignant features. Based on these conventional ultrasound features, it is difficult to distinguish malignant from benign. Further, inspection is needed, such as FNA. However, we know that FNA also has its limitations in identifying benign and malignant thyroid nodules, such as traumatic, unsatisfactory selection of materials, and uncertainty of results. That will cause mental and economic pressure to patients and clinical confusion to doctors. Except for conventional ultrasound indicators, is there a simple, noninvasive way to provide doctors with more diagnostic information and help assess the benign and malignant of thyroid nodules?

US real-time elasticity imaging technology has become well-developed in recent years, showing superiority for breast, thyroid, and prostate tissue, among others, due to its capacity for indicating tissue hardness [7–9]. In the clinical evaluation of thyroid nodules, the increased hardness of a nodule is closely associated with an increased risk of malignancy. The elasticity score (ES) of real-time tissue elastography (RTE) can reflect the elasticity characteristic (hardness) of tissue, while tissue hardness is closely associated with its internal pathological structure. Malignant nodules have higher tissue hardness as compared with benign nodules, and reconstruction of tissue elasticity could provide important information for the diagnosis of the disease [10, 11]. Some studies have evaluated the diagnostic performance of RTE or the combination of RTE and US. However, most studies were performed in small series and evaluated RTE only without comparison with grayscale US, or just compared with combinations of a few suspicious grayscale US features [12–14]. The 2017 ACR TI-RADS thyroid guidelines did not make regular recommendations for RTE in thyroid nodule assessment, mainly due to inconsistent results of RTE reported by previous studies [15]. Nevertheless, Tian et al. [16] conducted a meta-analysis of the use of RTE to differentiate malignant and benign thyroid nodules, which showed a good sensitivity of 0.787 and a specificity of 0.805. Hence, the adjunctive performance of RTE remains unclear.

Therefore, we aimed to investigate the correlation between the ES of RTE and the malignant risk stratification of ACR TI-RADS category and to evaluate the added value of RTE to TI-RADS in differentiating malignant nodules from benign ones.

## 2. Materials and Methods

This retrospective study was approved by our ethics committee, which waived the requirement for informed patient consent because the data for all subjects were anonymized.

### 2.1. Patients and Nodules

A total of 1,498 patients involving 1,525 thyroid nodules who underwent FNA and/or thyroidectomy in our hospital between May 2017 and August 2019 were included for analysis. The patients comprised 613 men and 885 women, with a mean age of 43.5 ± 12.4 years ranging from 14 to 85 years. The inclusion criteria were as follows: (1) largest nodule diameter up to 2.5 cm; (2) no previous surgery or US-guided percutaneous thermotherapy; (3) underwent conventional US and RTE within one month before FNA and/or thyroidectomy. Cases were excluded if (1) they had coalescent thyroid lesions that were not clearly distinguishable; (2) they had nodules with ambiguous FNA diagnosis; (3) information about nodule was incomplete.

### 2.2. Real-Time Grayscale US Assessment of Thyroid Nodules

HI Vision 900, HI Vision Ascendus, and HI Vision Preirus colour US units (with US elasticity imaging capability) from Hitachi were used for RTE, and the probe frequency was 6.0–13.0 MHz. The following features were measured for each nodule: size, composition, echogenicity, margin, shape, and echogenic foci. The classifications and definitions of nodule features are shown in Table 1.

### 2.3. ACR Thyroid Imaging Reporting and Data System

The TI-RADS classification of nodules was conducted according to the 2017 ACR management guidelines [17]. According to the TI-RADS system, five features of each nodule were evaluated, including (1) component (choose one): cystic or almost completely cystic 0; spongiform 0; mixed cystic and solid 1; solid or almost completely solid 2; (2) echogenicity (choose one): anechoic 0; hyperechoic or isoechoic 1; hypoechoic 2; very hypoechoic 3; (3) shape (choose one): wider-than-tall, 0; taller-than-wide, 3; (4) margin (choose one): smooth 0; Ill-defined 0; lobulated or irregular 2; extrathyroid extension 3; and (5) echogenic foci (choose one): none or large comet-tail artifact 0; macrocalcification 1; peripheral (rim) calcification 2; punctate echogenic foci 3. Each feature was assigned a corresponding score, and then the nodules were assigned to different TI-RADS categories according to their total scores. Nodules with score 0 were classified as TI-RADS category 1 (malignancy risk of 2%, benign), score 2 were classified as TI-RADS category 2 (malignancy risk of 2%, probably benign), score 3 were classified as TI-RADS category 3 (malignancy risk of 5%, mild suspicious malignancy), score 4 to 6 were classified as TI-RADS category 4 (malignancy risk of 5–20%, moderate suspicious malignancy), and score ≥7 were classified as TI-RADS category 5 (malignancy risk of >20%, highly suspicious malignancy).

### 2.4. US Real-Time Strain Elastography

HI Vision 900, HI Vision Ascendus, and HI Vision Preirus colour US units (with US elasticity imaging capability) from Hitachi were used for strain elastography, and the probe frequency was 6.0–13.0 MHz. US elastography was routinely included in the thyroid US for all patients in the authors' institution. The conventional US and strain elastography were performed by two radiologists on the same day. The criteria for reliable elasticity imaging were as follows [15, 18]. The handheld probe made a gentle vibration at the lesion site. A square region of interest positioning the target nodule at the centre of the box was set for elastography acquisition. The superior and inferior margins were set to include subcutaneous fat and longus colli muscle, respectively. The pressure release index on the instrument display was maintained between 3 and 4. Elastic images that clearly reflect tissue hardness in each layer were considered as appropriate. The imaging lasted for 3–5 s.

Different colour reflects different relative hardness levels in the lesion area. If more than 50% of the lesion was shown as green, it was defined as green-based, and if more than 50% was shown as blue, it was blue-based. The elastography performance was categorized into scores 0–4 based on the Asteria criteria as follows [18–20]: 0, the lesion region mainly contained cystic component manifested as alternating red and blue or alternating blue, green, and red, which presented a typical “mosaic” sign; 1, both the lesion and surrounding tissues showed a consistent green colour; 2, the lesion region showed an alternating blue and green colour with green as the main colour; 3, the lesion region had an alternating blue and green colour with blue as the main colour; and 4, the lesion region was completely covered by blue colour. In elastography, red represents soft, blue represents hard, and alternating from red to green to blue represents the process from soft to hard. In the elastic diagram of nodules, the more red and green the components, the softer the nodules and the lower the score; the more blue, the harder the nodules and the higher the score.

The ACR TI-RADS and RTE were performed by two radiologists with more than 10 years of experience in the diagnosis of thyroid disease. Both radiologists are experienced with developing the diagnostic criteria of TI-RADS and the ES system. In case of inconsistent opinions, a conclusion was reached by discussion with a third radiologist.

## 3. Statistical Analysis

Statistical analyses were performed by SPSS version 20.0 (SPSS Inc., Chicago, Illinois) and Stata version 11.0 (StataCorp LP, Texas). Spearman's rank correlation was used to evaluate the correlation between the ES of RTE and the malignant risk stratification of the TI-RADS category. Binary logistic regression based on two variables (ES and TI-RADS category) was conducted to generate a logistic regression equation of malignancy prediction probability: Logit (*P*) = *α*ES + *β* (TI-RADS category) + constant, where *α* and *β* were the regression coefficients. The combined predictor (ES plus TI-RADS category) was then calculated as the TI-RADS category +*α*/*β* ES. Receiver operator characteristic (ROC) analyses, including sensitivity, specificity, and area under the curve (AUC), which were performed to evaluate the diagnostic performance of RTE, TI-RADS, and combined predictor. FNA or postoperative pathology was used as a reference. A *P* value of <0.05 was considered to be statistically significant.

## 4. Results

### 4.1. Patient and Nodule Characteristics

In our study, there were 1,525 nodules in 1498 patients (including 1470 patients with one nodule, 26 patients with two nodules, one patient with three nodules, respectively). Of the 1525 nodules, 726 were benign (673 were nodular goiter, 47 adenoma, and 7 inflammation) and 799 were malignant (785 were papillary carcinoma, 4 follicular carcinoma, 7 medullary carcinoma, and 3 undifferentiated carcinoma). 501 benign and 724 malignant nodules were confirmed with surgery, and 225 benign and 75 malignant were confirmed with the cytologic examination.

Among them, 75 malignant confirmed by cytology had undergone surgery and were confirmed by surgery finally. Of the 1,525 nodules, a total of 134 nodules were classified as TI-RADS 4. Among these 134 cases were of TI-ADS 4 nodules, 72 cases were benign, and 62 cases were malignant, confirmed by pathology. The mean age of patients was associated with nodule malignancy (*P* < 0.001). The number of female patients with malignant nodules was higher than that of male patients (*P* < 0.001) (Table 2).

Among the 1525 thyroid nodules evaluated by two radiologists, 1522 of which were consistent between the two radiologists, but there were some differences in the evaluation of three nodules (two nodules, one radiologist thought that their RTE should be score 4, but the other one thought that they should be score 3. After the third radiologist evaluates repeatedly, the final score was 3. The remaining nodule, one radiologist thought it should be score 3, but the other one thought it should be score 2. After the third radiologist evaluates repeatedly, the final score was 3.) There was no significant difference between the two radiologists (*P* < 0.001).

### 4.2. Grayscale US Features, RTE, and TI-RADS Associated with Malignancy

Conventional grayscale US features of hypoechogenicity and very hypoechoic, poorly defined margin, taller-than-wide, Punctate echogenic foci, and Punctate echogenic foci plus macrocalcification were more common in malignant nodules (for all, *P* < 0.001). Scores 3 and 4 based on the Asteria criteria were also more common in malignant nodules (*P* < 0.001). Malignant nodules were mainly located in TI-RADS 4 and 5, particularly in TI-RADS 5 (for all, *P* < 0.001) (Table 3).

### 4.3. Correlation between the ES and TI-RADS in Various Size Intervals

Overall, a strong correlation was found between the ES (0–4) and the malignant risk stratification (1–5) of TI-RADS category in the assessment of thyroid nodules. The two methods showed a strong correlation in the nodule size intervals of 0.5 < *D* ≤ 1.0 cm (*r* = 0.768, *P* < 0.001), 1.0 < *D* ≤ 2.0 cm (*r* = 0.711, *P* < 0.001), and 2.0 < *D* ≤ 2.5 cm (*r* = 0.743, *P* < 0.001). However, the correlation was low (*r* = 0.481, *P* < 0.001) in nodules with a maximum diameter <0.5 cm.

### 4.4. Diagnostic Performance of the RTE and TI-RADS in Various Size Intervals

**Figure 1:**
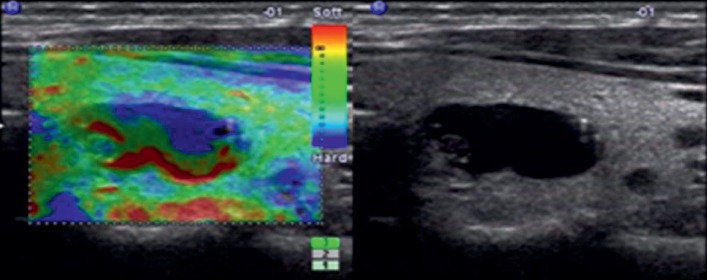
Images in a 28-year-old woman who underwent routine check-up. A 16 mm left thyroid nodule with no echo, cystic, smooth margin, wider-than-tall, and no calcification was found by grayscale US and assessed as a benign nodule. A score of 0 was assigned at elastography. The TI-RADS category was assigned as 1. This thyroid nodule was diagnosed as a nodular goiter with cystic change at surgery.

**Figure 2:**
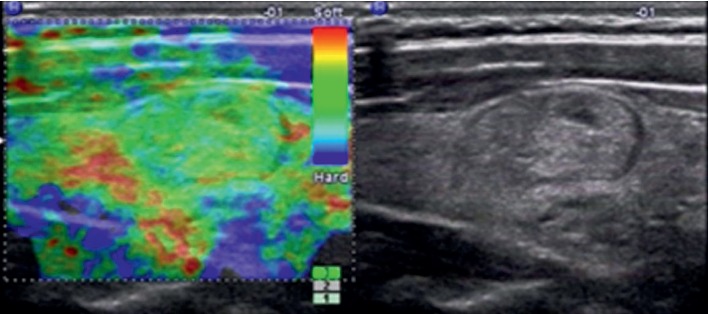
Images in a 35-year-old woman who underwent routine check-up. A 14 mm right thyroid nodule with isoechogenicity, solid, smooth margin, wider-than-tall, and no calcification was found by grayscale US and assessed as a benign nodule. A score of 1 was assigned at elastography. The TI-RADS category was assigned as 2. This thyroid nodule was diagnosed as a nodular goiter at surgery.

**Figure 3:**
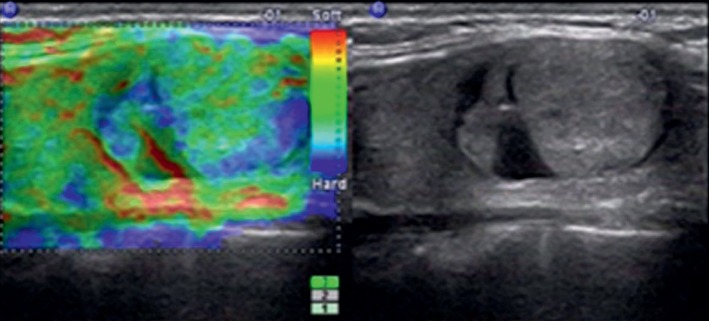
Images in a 41-year-old man who underwent routine check-up. A 20 mm left thyroid nodule with isoechogenicity, mixed, smooth margin, wider-than-tall, and no calcification was found at grayscale US and assessed as a benign nodule. A score of 2 was assigned at elastography. The TI-RADS category was assigned as 3. This thyroid nodule was diagnosed as nodular goiter at surgery.

**Figure 4:**
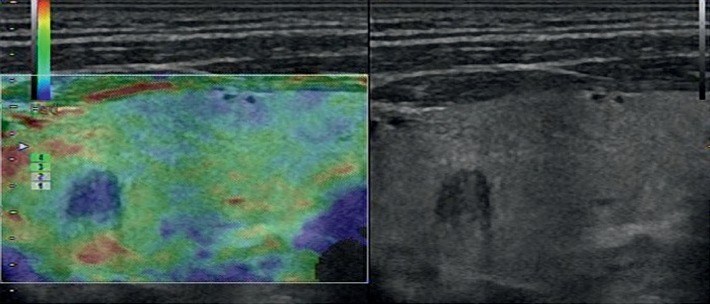
Images in a 42-year-old woman who underwent routine check-up. A 6 mm left thyroid nodule with hypoechogenicity, solid, irregular margins, wider-than-tall, and no calcification was found by conventional US and assessed as a benign nodule. A score of 3 was assigned at elastography. The TI-RADS category was assigned as 4. This thyroid nodule was diagnosed as papillary thyroid carcinoma at surgery.

**Figure 5:**
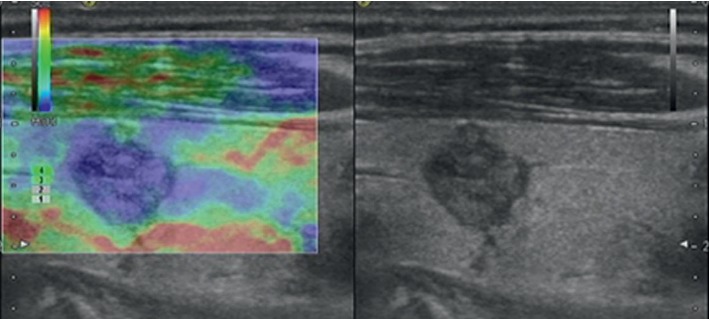
Images in a 65-year-old woman who underwent routine check-up. A 10 mm left thyroid nodule with hypoechogenicity, solid, irregular margin, taller-than-wide, and no calcification was found by grayscale US and assessed as a malignant nodule. A score of 4 was assigned at elastography. The TI-RADS category was assigned as 5. This thyroid nodule was diagnosed as papillary thyroid carcinoma at surgery.

The sensitivity, specificity, and AUC of the TI-RADS category values were significantly higher than those of the ES system of RTE in nodules with *D* ≤ 0.5 cm. In contrast, the sensitivity, specificity, and AUC of the ES system of RTE were significantly higher than those of TI-RADS in nodules with 0.5 < *D* ≤ 2.0 cm (Table 4) (Figures 1–5).

**Figure 6:**
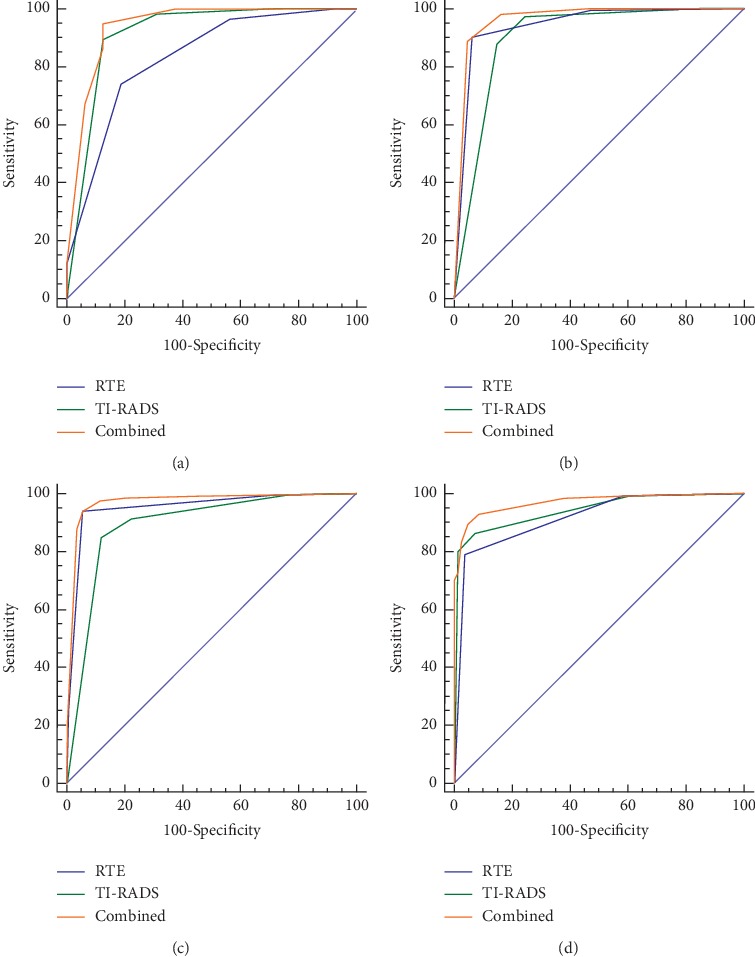
ROC analyses of RTE, TI-RADS, and RTE combined TI-RADS in the size intervals of *D* ≤ 0.5 cm (a), 0.5 < *D* ≤ 1.0 cm (b), 1.0 < *D* ≤ 2.0 cm (c), and 2.0 < *D* ≤ 2.5 cm (d).

The ROC curve showed that the combination of the two methods gave a higher AUC as compared with either the ES system of RTE or TI-RADS alone, including nodules of *D* ≤ 0.5 cm, 0.5 < *D* ≤ 1.0 cm, 1.0 < *D* ≤ 2.0 cm, and 2.0 < *D* ≤ 2.5 cm (for all, *P* < 0.001) (Figure 6). The sensitivity of the combined method was significantly higher than that of TI-RADS (*P* < 0.001) (Table 4). The specificity of the combined method was also significantly higher than that of TI-RADS in each size interval (for all, *P* < 0.001) (Table 4).

## 5. Discussion

Here, we retrospectively analyzed US and RTE data of 1525 thyroid nodules. We found that the ES of RTE and the malignant risk stratification of TI-RADS showed a strong correlation in the nodule size intervals of 0.5 < *D* ≤ 1.0 cm, 1.0 < *D* ≤ 2.0 cm, and 2.0 < *D* ≤ 2.5 cm, except for the interval of *D* ≤ 0.5 cm. The diagnostic performance of the combination of RTE and TI-RADS in each size interval was always better than either RTE or TI-RADS alone. These results demonstrated the feasibility of strengthening the diagnosis of malignant thyroid nodules by RTE.

The ROC analysis showed RTE had higher AUCs in the size intervals of 0.5 < *D* ≤ 1.0 cm and 1.0 < *D* ≤ 2.0 cm than in the intervals of *D* ≤ 0.5 cm and 2.0 < *D* ≤ 2.5 cm, which indicated that RTE may be more suitable for applying in nodules with size between 0.5 and 2.0 cm. For those nodules smaller than 0.5 cm or larger than 2.0 cm, the force applied during the RTE process was more likely to be uneven. To maintain even pressure on a nodule requires each part of the lesion and the surrounding gland tissues being deformed under the same pressure to obtain an accurate elasticity score. Maintaining even pressure on larger nodules is difficult because of the increased tension and inadequate deformation caused by a long course of the disease and cystic changes within nodules, which would result in artificially higher RTE scores. For nodules smaller than 0.5 cm, the hardness is similar to the surrounding normal thyroid tissues due to genetic and biological heterogeneity, unnoticeable internal fibrosis hyperplasia, and no accompanying calcifications [21]. Thus, the elastography results were less satisfactory for nodules smaller than 0.5 cm or larger than 2.0 cm.

RTE could overcome the limitations of conventional detection techniques. It is widely used to distinguish malignant from benign nodules based on the elasticity or stiffness of the tissues [22–28]. We found that scores 3 and 4 were more common in malignant nodules, which was in accordance with the results of previous studies [29, 30]. However, the diagnostic value of RTE did not gain recognition in several studies [15, 20, 31–34]. One study of 703 thyroid nodules found that RTE led to lower diagnostic accuracy than US, and one prospective study showed that the positive predictive value of elastography was only 36%, and the negative predictive value was 97%. The variation was mainly due to discrepancies in nodule selection, different equipment, and the experience of operators [35]. The tissue hardness assessed by RTE was relative hardness, which is the ratio of hardness between the lesion and surrounding normal tissues. This assessment required normal tissues surrounding the lesion for comparison to obtain a good imaging result. Therefore, we selected nodules ≤2.5 cm for assessment, which was in line with the findings of Veyrieres et al. that RTE cannot be applied to nodules larger than 3 cm [36]. In our institution, RTE was performed under common criteria to ensure the accuracy of the elasticity score system. This study was aimed to explore the additional value of RTE to the TI-RADS. The TI-RADS system referred to in the 2017 ACR guidelines had a satisfactory effect on the assessment of malignancy risk of thyroid nodules. TI-RADS played an important role in guiding clinically standardized treatments and improving the diagnosis and treatment levels. This study showed that TI-RADS performed well in the evaluation of malignant thyroid nodules in all size intervals, with AUCs of 0.917, 0.918, 0.905, and 0.938 for the four size intervals. In addition, after adding RTE to the TI-RADS system, the sensitivity, specificity, and AUC of TI-RADS were significantly improved. In addition, in our study, a total of 134 nodules were classified as TI-RADS 4, and 72 benign and 62 malignant nodules were confirmed by pathology. Among the 134 TI-RADS 4 nodules, RTE accurately diagnosed 41 malignant and 70 benign, the sensitivity, specificity, and accuracy of RTE in the diagnosis of such nodules were 97.2%, 66.1%, and 82.8%, respectively. These results suggested that RTE could provide additional information for TI-RADS, thereby improving its diagnostic accuracy for malignant thyroid nodules. The elasticity score reflects tissue hardness based on the pathological structural characteristics of a nodule. For example, fibrosis-associated hyperplasia and sand-like calcifications within the malignant nodules resulted in increased tissue hardness, which was assigned a score 3 or 4 corresponding to TI-RADS 5. In contrast, the hardness of benign nodules was similar to the surrounding normal thyroid tissues because the only hyperplasia of thyroid tissue was found in the benign nodules, so they were assigned scores 1 or 2 corresponding to TI-RADS 2 or 3. Therefore, the ES of RTE and the malignant risk stratification of TI-RADS demonstrated good consistency for benign and malignant pathological features.

Currently, there are two main elastographic systems available on the market: strain elastography and shear wave elastography (SWE). In SWE, shear wave emission is induced by a focused ultrasonic beam. The elasticity of the tissue is assessed qualitatively and quantitatively in real-time based on the received signals, which is considered to be more objective, reliable, and reproducible than RTE [36–39]. According to Zhang et al. [40], the sensitivity, specificity, and AUC for the diagnosis of malignant thyroid nodules by SWE were 0.84, 0.90, and 0.92, respectively. Zhuo et al. [41] and Sebag et al. [42] reported that the sensitivity and specificity for the diagnosis of malignant thyroid nodules by SWE were 0.963 and 0.962, and 0.852 and 0.939, respectively. However, SWE also has some disadvantages. Sebag et al. reported that macrocalcifications gave a high false-positive rate for malignancy when using SWE [42]. For microcarcinomas with a maximum diameter of ≤1 cm, the diagnostic performance of SWE was not satisfactory. The majority of the microcarcinomas were missed by SWE [27]. In the present study, we employed a strain elastography system to evaluate the hardness of thyroid nodules. It did not have the disadvantages of SWE as mentioned above. It could better find the nodules with a diameter less than or equal to 1 cm and reflect the hardness change caused by microcalcification and fibrosis-associated hyperplasia inside the nodules.

In our research, the malignancy rate was higher for TI-RADS 3, 4, and 5 categories than expected. This may be related to the cases we have collected. Affected by the level of ultrasound diagnosis, we included some atypical nodules that were difficult to be classified by ACR TI-RADS, and more typical ACR TI-RADS 5 nodules. In addition, this study also has some other limitations. Firstly, we selected only nodules smaller than 2.5 cm because the size range was suitable for elastography. Secondly, among malignant nodules, papillary carcinoma was the majority, and the number of other types of carcinoma was few. Studies to analyze more other types of malignant nodules are anticipated.

## 6. Conclusions

In conclusion, there is a strong correlation between the ES system of RTE and the malignant risk stratification of the TI-RADS classification system, the ES system of RTE improves the diagnostic performance of the ACR TI-RADS in the evaluation of malignancy risk of thyroid nodules, especially for TI-RADS 4 nodules. RTE is recommended as an adjunctive diagnostic tool to ACR TI-RADS for differentiating malignant from benign thyroid nodules.

## Figures and Tables

**Table 1:** The classifications and definitions of nodule features.

Features	Classifications	Definitions
Size intervals	*D* ≤ 0.5 cm, 0.5 < *D* ≤ 1.0 cm, 1.0 < *D* ≤ 2.0 cm, 2.0 < *D* ≤ 2.5 cm	Maximum diameter of the nodule

Composition	Cystic/sponge-like/mixed/solid	Cystic: the complete composition of the nodules is cystic/sponge-like: composed of multiple microcystic components, accounting for more than 50% of the nodule volume/mixed: solid and cystic components are >5%/solid nodules: solid components >95%

Echogenicity	Absence/hyperechogenicity or isoechogenicity/hypoechogenicity/very hypoechogenicity	Absence: for cystic or almost complete cystic nodules/hyperechogenicity or isoechogenicity/hypoechogenicity: reference is normal thyroid tissue next to the nodule/very hypoechogenicity: reference is adjacent to the anterior cervical muscle

Margin	Smooth/ill-defined/lobulated or irregular/extrathyroidal extension	Smooth: the boundary between the nodule and the parenchyma of the gland is clear/ill-defined: not clear/lobulated or irregular: the dividing line between nodule and surrounding thyroid parenchyma is irregular/extrathyroidal extension: the capsule of thyroid is incomplete and the continuity is interrupted

Shape	Tall/wide <1/tall/wide >1	Wider-than-tall/taller-than-wide

Echogenic foci pattern	None or large comet-tail artifacts/macrocalcification/peripheral (rim) calcifications/punctate echogenic foci	Large comet-tail artifacts: artifact depth >1 cm, present in cystic composition/coarse calcification: rear with sound shadow/peripheral calcification: calcification completely or partially encircles most of the edge of the nodule/punctate echogenic foci: no sound behind, diameter <1 cm, including the small hui tail sign in the solid composition

**Table 2:** Nodule size and numbers of nodules and patients.

Parameter	Nodules	Patients	Final diagnosis		
			Benign	Malignant	*P* value^*∗*^
All nodules	1525	1498	726	799	
Mean patient age (y)		43.5	44.7	42.3	<0.001
Women	904	887	352	552	<0.001
Men	621	611	374	247	
0 < *D* ≤ 0.5 cm	74	71	16	58	
Mean patient age (y)		44.4	45.6	44.1	0.625
Women	47	44	5	42	0.002
Men	27	27	11	16	
0.5 < *D* ≤ 1.0 cm	528	518	197	331	
Mean patient age (y)		43.0	43.1	42.9	<0.815
Women	315	308	80	235	<0.001
Men	213	210	117	96	
1.0 < *D* ≤ 2.0 cm	715	704	429	286	
Mean patient age (y)		44.3	45.1	43.1	0.035
Women	426	417	228	198	<0.001
Men	289	287	201	88	
2.0 < *D* ≤ 2.5 cm	208	205	84	124	
Mean patient age (y)		41.7	46.6	38.4	<0.001
Women	116	116	39	77	0.026
Men	92	89	45	47	

*Note.* Unless otherwise indicated, the data point is the number of nodules or patients. ^*∗*^*P* value calculated by using the generalized estimating equation analysis.

**Table 3:** Comparison of conventional ultrasound features, real-time strain elastography scores, and TI-RADS categories between the benign and malignant nodules.

Features	No. of benign nodules (*n* = 726)	No. of malignant nodules (*n* = 799)	*P* value^*∗*^
Composition			<0.001^#^
Cystic (*n* = 15)	15 (2.1)	0	<0.001
Spongiform (*n* = 16)	16 (2.2)	0	<0.001
Mixed cystic and solid (*n* = 97)	71 (9.8)	26 (3.3)	<0.001
Solid or almost completely solid (*n* = 1,397)	624 (85.9)	773 (96.7)	<0.001
Echogenicity			<0.001^#^
Absence (*n* = 31)	31 (4.3)	0	<0.001
Hyperechoic or isoechogenicity (*n* = 600)	498 (68.5)	102 (12.8)	<0.001
Hyperechogenicity (*n* = 61)	61	0	
Hypoechogenicity (*n* = 863)	193 (26.6)	670 (83.8)	<0.001
Very hypoechoic (*n* = 31)	4 (0.6)	27 (3.4)	<0.001
Margin			<0.001
Smooth (*n* = 687)	573 (78.9)	114 (14.3)	
ill-defined (*n* = 597)	129 (17.8)	468 (58.6)	
Lobulated or irregular (*n* = 52)	11 (1.5)	41 (5.1)	
Extrathyroidal ertension (*n* = 189)	13 (1.8)	176 (22.0)	
Shape			<0.001
Wider-than-tall (*n* = 1,101)	691 (95.2)	410 (51.3)	
Taller -than-wide (*n* = 424)	35 (4.9)	389 (48.7)	
Echogenic foci			<0.001^#^
None or large comet-tail (*n* = 766)	606 (83.5)	160 (20.0)	<0.001
Punctate echogenic foci (*n* = 547)	21 (2.9)	526 (65.8)	<0.001
Macrocalcifications (*n* = 122)	73 (10.1)	49 (6.1)	
Periphera (rim) calcifications (*n* = 21)	15 (2.1)	6 (0.8)	
Punctate + macrocalcification (*n* = 69)	11 (1.5)	58 (7.3)	<0.001
ES system of RTE			<0.001
Score 0 (*n* = 31)	31 (4.3)	0 (0)	
Score 1 (*n* = 241)	236 (32.5)	5 (0.6)	
Score 2 (*n* = 503)	417 (57.4)	86 (10.8)	
Score 3 (*n* = 639)	39 (5.4)	600 (75.1)	
Score 4 (*n* = 111)	3 (0.4)	108 (13.5)	
TI-RADS			<0.001
1 (*n* = 31)	31 (4.3)	0 (0)	
2 (*n* = 162)	159 (21.9)	3 (0.4)	
3 (*n* = 430)	381 (52.5)	49 (6.1)	
4 (*n* = 134)	72 (9.9)	62 (7.8)	
5 (*n* = 768)	83 (11.4)	685 (85.7)	

*Note.* Unless otherwise indicated, the data point is number of nodules, and the number in parentheses is percentage. ^*∗*^*P* value calculated by using the generalized estimating equation analysis. ^#^Fisher exact test.

**Table 4:** Diagnostic performance of RTE, TI-RADS, or both in 1525 nodules.

Size interval (cm)	AUC (95%CI)	Sensitivity (%)	Specificity (%)	Accuracy (%)	PPV (%)	NPV (%)
0 < *D* ≤ 0.5						
TI-RADS	0.916 (0.815–1.000)	89.7	87.5	91.89	91.94	91.67
RTE	0.832 (0.714–0.950)	74.1	81.2	75.67	93.48	46.43
TI-RADS plus RTE	0.941 (0.861–1.000)	94.8	87.5	93.24	96.49	82.35
0.5 < *D* ≤ 1.0						
TI-RADS	0.903 (0.871–0.935)	87.9	85.3	89.20	87.03	94.30
RTE	0.943 (0.920–0.966)	90.0	93.9	91.48	96.13	84.86
TI-RADS plus RTE	0.961 (0.941–0.982)	90.0	93.9	91.29	97.03	83.56
1.0 < *D* ≤ 2.0						
TI-RADS	0.894 (0.869–0.919)	85.0	88.1	83.08	73.11	93.02
RTE	0.954 (0.937–0.970)	94.1	94.4	94.27	91.81	95.97
TI-RADS plus RTE	0.972 (0.960–0.985)	94.3	95.6	94.41	92.12	95.98
2.0 < *D* ≤ 2.5						
TI-RADS	0.942 (0.912–0.973)	86.3	92.9	88.94	94.69	82.11
RTE	0.919 (0.881–0.956)	79.0	96.4	86.06	97.03	75.70
TI-RADS plus RTE	0.971 (0.951–0.990)	89.5	95.2	91.83	96.52	86.02

*Note.* Unless otherwise indicated, the data point is percentage, and the number in parentheses is 95% CI. PPV = positive predictive value; NPV = negative predictive value.

## Data Availability

The data sets used and/or analyzed during the current study are available from the corresponding author upon reasonable request.

## Abbreviations

ES: Elasticity score
RTE: Real-time elastography
TI-RADS: Thyroid imaging reporting and data system
CT: Computed tomography
MRI: Magnetic resonance imaging
PET: Positron emission tomography
ACR: American College of Radiology
ROC: Receiver operator characteristic
AUC: Area under the curve
CI: Confidence interval
PPV: Positive predictive value
NPV: Negative predictive value.
